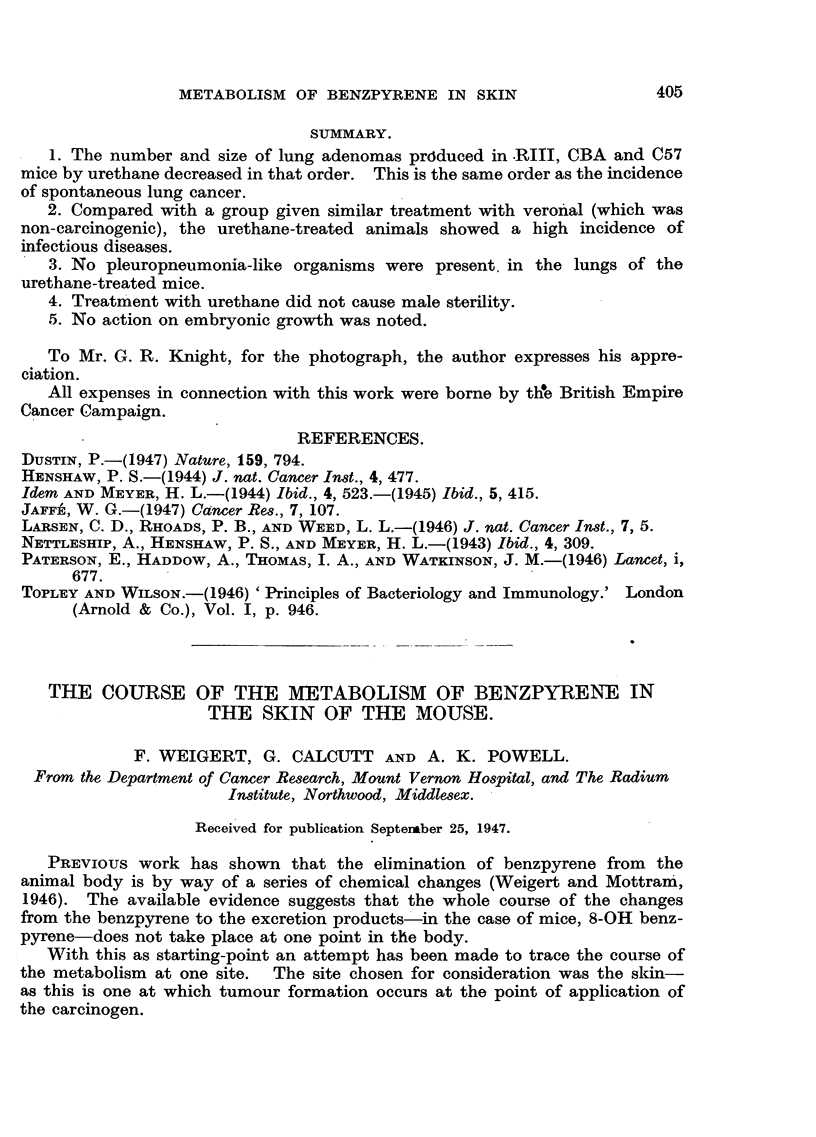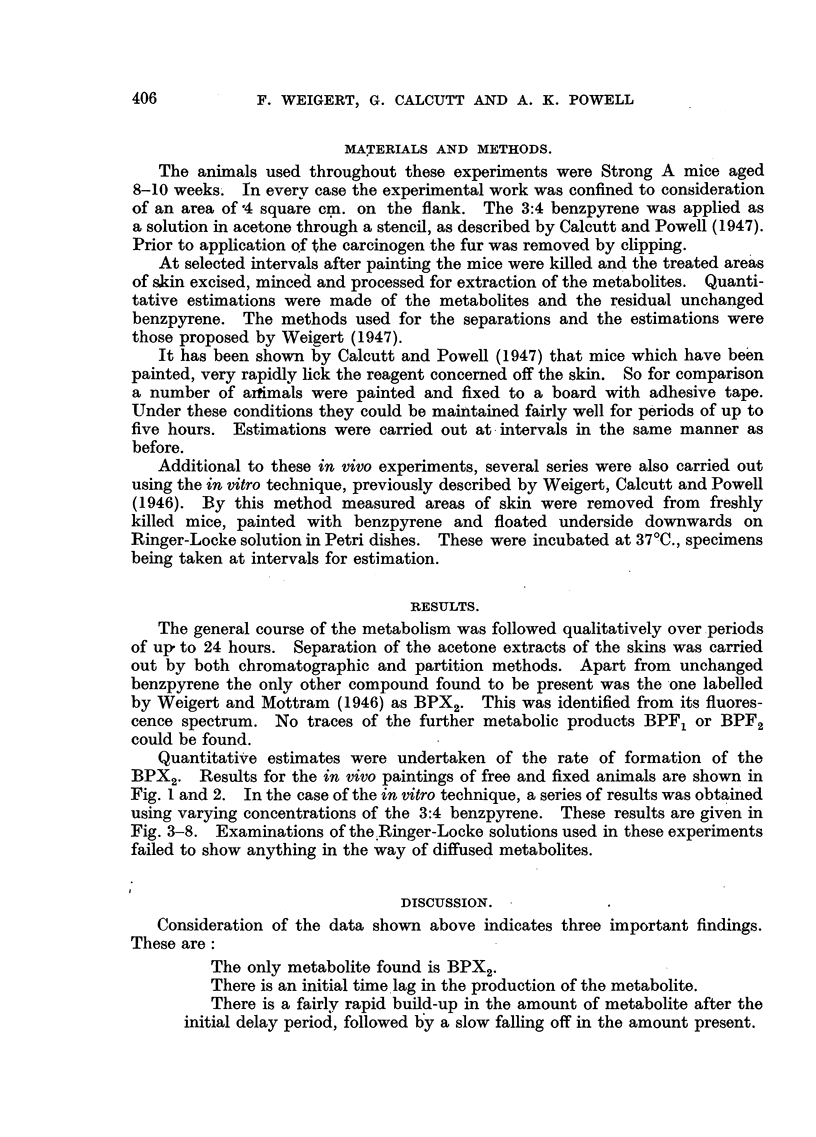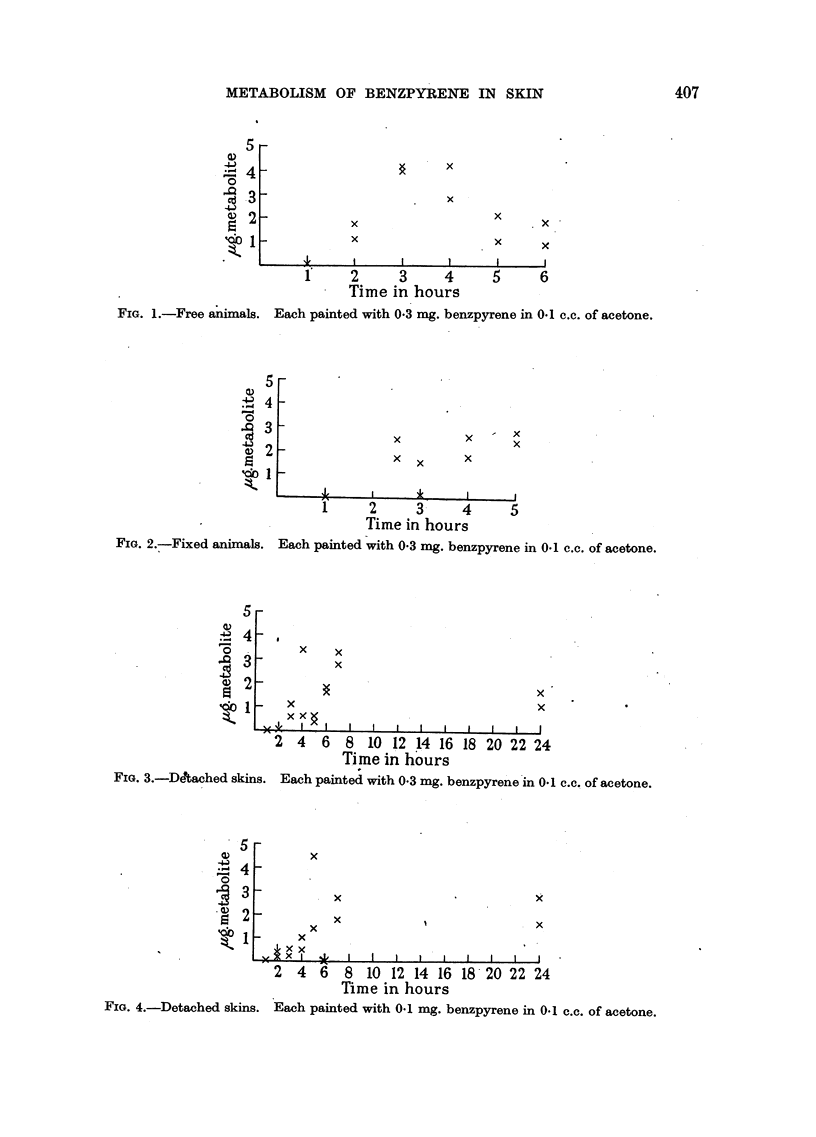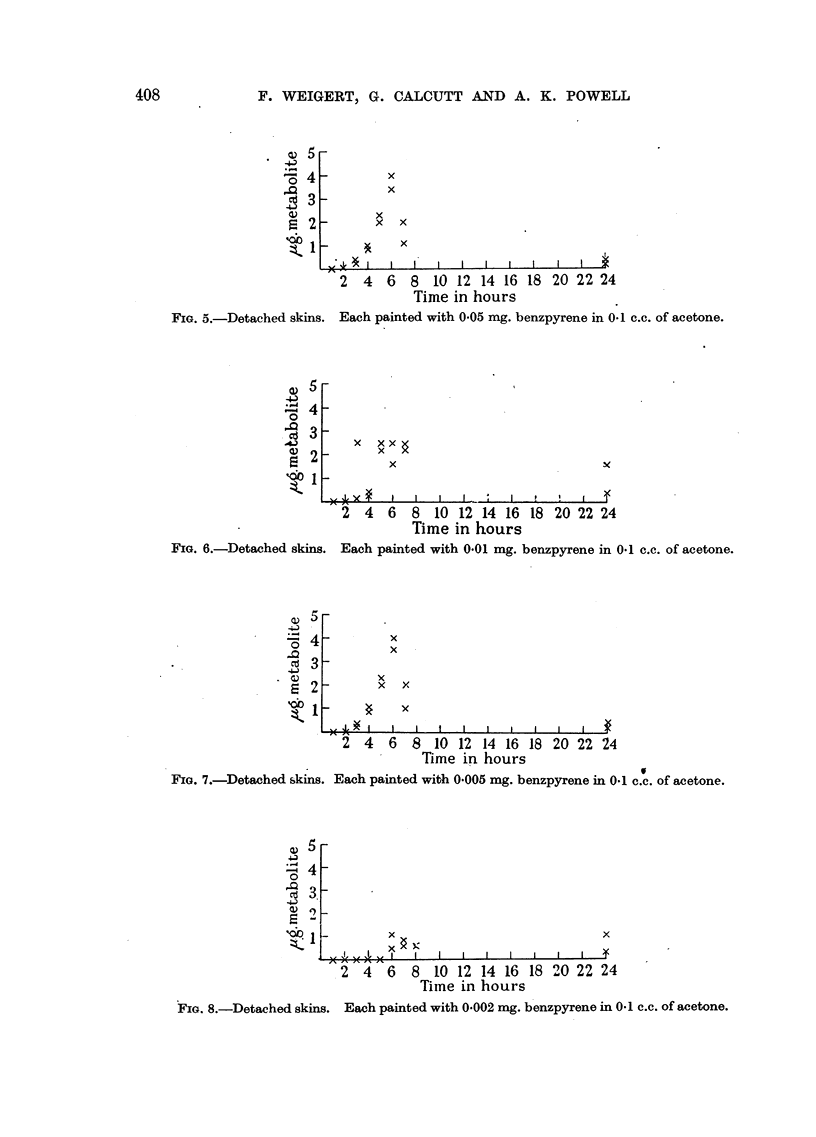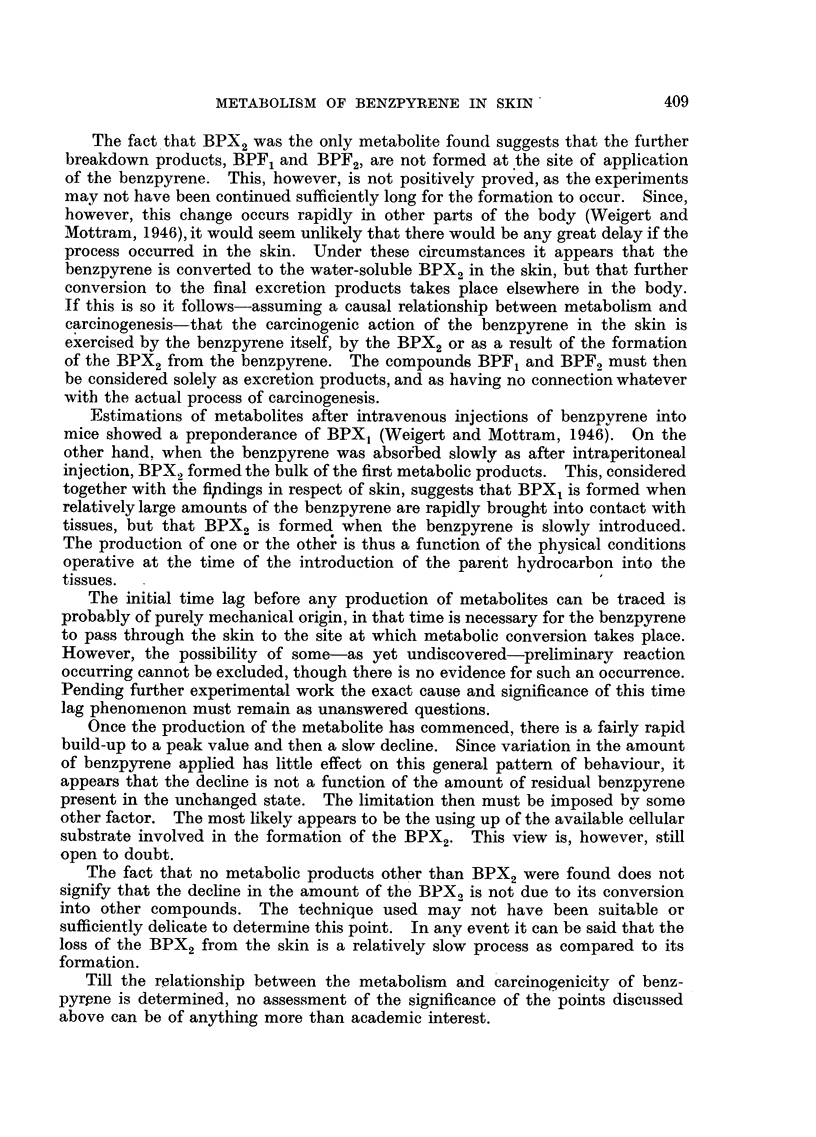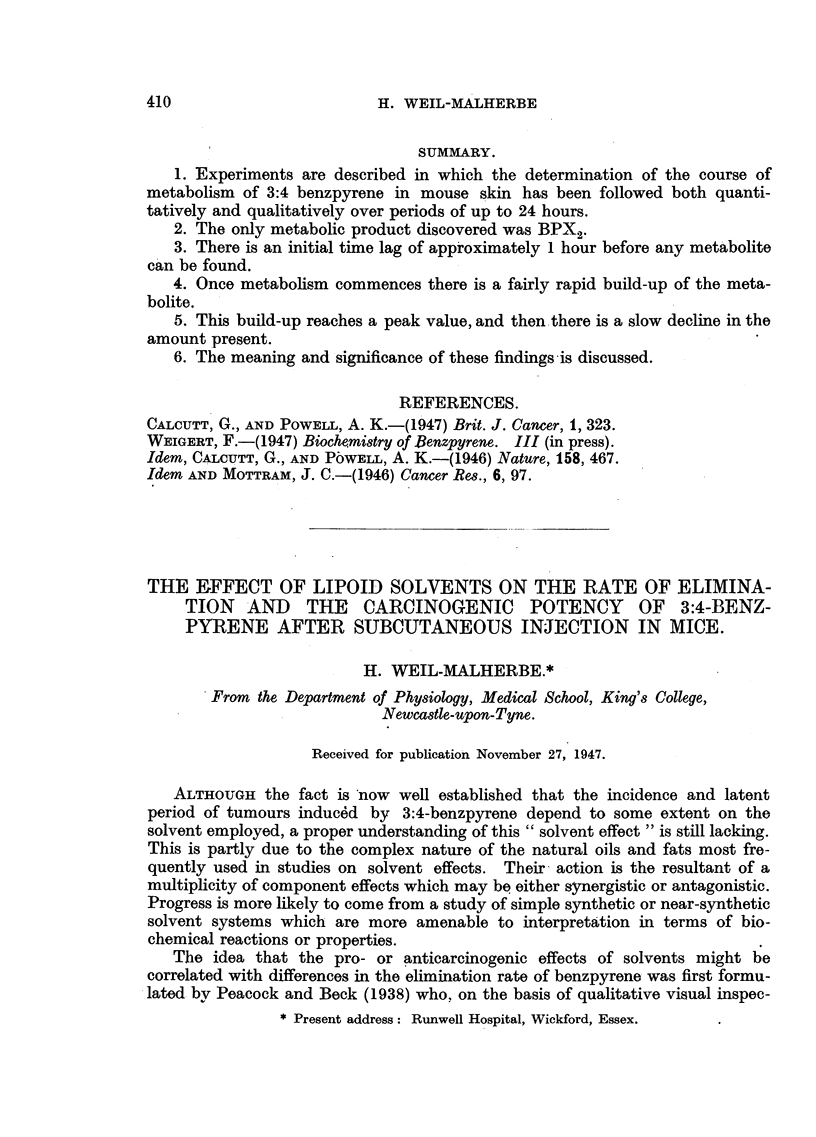# The Course of the Metabolism of Benzpyrene in the Skin of the Mouse

**DOI:** 10.1038/bjc.1947.40

**Published:** 1947-12

**Authors:** F. Weigert, G. Calcutt, A. K. Powell


REFERENCES.

Dustin, P.—(1947) *Nature*, **159**, 794.
Henshaw, P. S.—(1944) *J. nat. Cancer Inst.*, **4**, 477.
*Idem* and Meyer, H. L.—(1944) *Ibid.*, **4**, 523.—(1945) *Ibid.*, **5**, 415.
Jaffé, W. G.—(1947) *Cancer Res.*, **7**, 107.
Larsen, C. D., Rhoads, P. B., and Weed, L. L.—(1946) *J. nat. Cancer Inst.*, **7**, 5.
Nettleship, A., Henshaw, P. S., and Meyer, H. L.—(1943) *Ibid.*, **4**, 309.
Paterson, E., Haddow, A., Thomas, I. A., and Watkinson, J. M.—(1946) *Lancet*, i, 677.
Topley and Wilson.—(1946) 'Principles of Bacteriology and Immunology.' London (Arnold & Co.), Vol. I, p. 946.

### THE COURSE OF THE METABOLISM OF BENZPYRENE IN THE SKIN OF THE MOUSE.

F. WEIGERT, G. CALCUTT AND A. K. POWELL.

*From the Department of Cancer Research, Mount Vernon Hospital, and The Radium Institute, Northwood, Middlesex.*

Received for publication September 25, 1947.

Previous work has shown that the elimination of benzpyrene from the animal body is by way of a series of chemical changes (Weigert and Mottram, 1946). The available evidence suggests that the whole course of the changes from the benzpyrene to the excretion products—in the case of mice, 8-OH benzpyrene—does not take place at one point in the body.

With this as starting-point an attempt has been made to trace the course of the metabolism at one site. The site chosen for consideration was the skin—as this is one at which tumour formation occurs at the point of application of the carcinogen.

### MATERIALS AND METHODS.

The animals used throughout these experiments were Strong A mice aged 8–10 weeks. In every case the experimental work was confined to consideration of an area of ·4 square cm. on the flank. The 3:4 benzpyrene was applied as a solution in acetone through a stencil, as described by Calcutt and Powell (1947). Prior to application of the carcinogen the fur was removed by clipping.

At selected intervals after painting the mice were killed and the treated areas of skin excised, minced and processed for extraction of the metabolites. Quantitative estimations were made of the metabolites and the residual unchanged benzpyrene. The methods used for the separations and the estimations were those proposed by Weigert (1947).

It has been shown by Calcutt and Powell (1947) that mice which have been painted, very rapidly lick the reagent concerned off the skin. So for comparison a number of animals were painted and fixed to a board with adhesive tape. Under these conditions they could be maintained fairly well for periods of up to five hours. Estimations were carried out at intervals in the same manner as before.

Additional to these *in vivo* experiments, several series were also carried out using the *in vitro* technique, previously described by Weigert, Calcutt and Powell (1946). By this method measured areas of skin were removed from freshly killed mice, painted with benzpyrene and floated underside downwards on Ringer-Locke solution in Petri dishes. These were incubated at 37°C., specimens being taken at intervals for estimation.

### RESULTS.

The general course of the metabolism was followed qualitatively over periods of up to 24 hours. Separation of the acetone extracts of the skins was carried out by both chromatographic and partition methods. Apart from unchanged benzpyrene the only other compound found to be present was the one labelled by Weigert and Mottram (1946) as $BPX_2$. This was identified from its fluorescence spectrum. No traces of the further metabolic products $BPF_1$ or $BPF_2$ could be found.

Quantitative estimates were undertaken of the rate of formation of the $BPX_2$. Results for the *in vivo* paintings of free and fixed animals are shown in Fig. 1 and 2. In the case of the *in vitro* technique, a series of results was obtained using varying concentrations of the 3:4 benzpyrene. These results are given in Fig. 3–8. Examinations of the Ringer-Locke solutions used in these experiments failed to show anything in the way of diffused metabolites.

### DISCUSSION.

Consideration of the data shown above indicates three important findings. These are :

The only metabolite found is $BPX_2$.

There is an initial time lag in the production of the metabolite.

There is a fairly rapid build-up in the amount of metabolite after the initial delay period, followed by a slow falling off in the amount present.

FIG. 1.—Free animals. Each painted with 0·3 mg. benzpyrene in 0·1 c.c. of acetone.

FIG. 2.—Fixed animals. Each painted with 0·3 mg. benzpyrene in 0·1 c.c. of acetone.

FIG. 3.—Detached skins. Each painted with 0·3 mg. benzpyrene in 0·1 c.c. of acetone.

FIG. 4.—Detached skins. Each painted with 0·1 mg. benzpyrene in 0·1 c.c. of acetone.

FIG. 5.—Detached skins. Each painted with 0·05 mg. benzpyrene in 0·1 c.c. of acetone.

FIG. 6.—Detached skins. Each painted with 0·01 mg. benzpyrene in 0·1 c.c. of acetone.

FIG. 7.—Detached skins. Each painted with 0·005 mg. benzpyrene in 0·1 c.c. of acetone.

FIG. 8.—Detached skins. Each painted with 0·002 mg. benzpyrene in 0·1 c.c. of acetone.

The fact that $BPX_2$ was the only metabolite found suggests that the further breakdown products, $BPF_1$ and $BPF_2$, are not formed at the site of application of the benzpyrene. This, however, is not positively proved, as the experiments may not have been continued sufficiently long for the formation to occur. Since, however, this change occurs rapidly in other parts of the body (Weigert and Mottram, 1946), it would seem unlikely that there would be any great delay if the process occurred in the skin. Under these circumstances it appears that the benzpyrene is converted to the water-soluble $BPX_2$ in the skin, but that further conversion to the final excretion products takes place elsewhere in the body. If this is so it follows—assuming a causal relationship between metabolism and carcinogenesis—that the carcinogenic action of the benzpyrene in the skin is exercised by the benzpyrene itself, by the $BPX_2$ or as a result of the formation of the $BPX_2$ from the benzpyrene. The compounds $BPF_1$ and $BPF_2$ must then be considered solely as excretion products, and as having no connection whatever with the actual process of carcinogenesis.

Estimations of metabolites after intravenous injections of benzpyrene into mice showed a preponderance of $BPX_1$ (Weigert and Mottram, 1946). On the other hand, when the benzpyrene was absorbed slowly as after intraperitoneal injection, $BPX_2$ formed the bulk of the first metabolic products. This, considered together with the findings in respect of skin, suggests that $BPX_1$ is formed when relatively large amounts of the benzpyrene are rapidly brought into contact with tissues, but that $BPX_2$ is formed when the benzpyrene is slowly introduced. The production of one or the other is thus a function of the physical conditions operative at the time of the introduction of the parent hydrocarbon into the tissues.

The initial time lag before any production of metabolites can be traced is probably of purely mechanical origin, in that time is necessary for the benzpyrene to pass through the skin to the site at which metabolic conversion takes place. However, the possibility of some—as yet undiscovered—preliminary reaction occurring cannot be excluded, though there is no evidence for such an occurrence. Pending further experimental work the exact cause and significance of this time lag phenomenon must remain as unanswered questions.

Once the production of the metabolite has commenced, there is a fairly rapid build-up to a peak value and then a slow decline. Since variation in the amount of benzpyrene applied has little effect on this general pattern of behaviour, it appears that the decline is not a function of the amount of residual benzpyrene present in the unchanged state. The limitation then must be imposed by some other factor. The most likely appears to be the using up of the available cellular substrate involved in the formation of the $BPX_2$. This view is, however, still open to doubt.

The fact that no metabolic products other than $BPX_2$ were found does not signify that the decline in the amount of the $BPX_2$ is not due to its conversion into other compounds. The technique used may not have been suitable or sufficiently delicate to determine this point. In any event it can be said that the loss of the $BPX_2$ from the skin is a relatively slow process as compared to its formation.

Till the relationship between the metabolism and carcinogenicity of benzpyrene is determined, no assessment of the significance of the points discussed above can be of anything more than academic interest.

SUMMARY.

1. Experiments are described in which the determination of the course of metabolism of 3:4 benzpyrene in mouse skin has been followed both quantitatively and qualitatively over periods of up to 24 hours.

2. The only metabolic product discovered was $BPX_2$.

3. There is an initial time lag of approximately 1 hour before any metabolite can be found.

4. Once metabolism commences there is a fairly rapid build-up of the metabolite.

5. This build-up reaches a peak value, and then there is a slow decline in the amount present.

6. The meaning and significance of these findings is discussed.

### REFERENCES.

Calcutt, G., and Powell, A. K.—(1947) *Brit. J. Cancer*, **1**, 323.
Weigert, F.—(1947) *Biochemistry of Benzpyrene. III* (in press).
*Idem*, Calcutt, G., and Powell, A. K.—(1946) *Nature*, **158**, 467.
*Idem* and Mottram, J. C.—(1946) *Cancer Res.*, **6**, 97.